# Supplementary material for: Female-dominated disciplines have lower evaluated research quality and funding success rates, for men and women
Source: eLife. 2024 Sep 5;13:RP97613. doi: 10.7554/eLife.97613 (PMC11377033; doi:10.7554/eLife.97613)
Supplement: Supplementary file 1. [file elife-97613-supp1.docx]

**Supplementary file 1: Table S1** **comparative descriptions of each of the 4 datasets**

|  | **Scoring research excellence** | | **Success in research funding** | | |
| --- | --- | --- | --- | --- | --- |
| **Country(ies)** | Aotearoa-NZ (PBRF) |  | Australia  ARC | EU  EIGE |  |
| **Subnational dataset** |  | UC |  |  | Canada  CIHR |
|  |  |  |  |  |  |
| **Discipline groupings** | 42 (43 in 2018) | 43 | 22 | 8 | 4 |
| **Measures of gender balance** | From national PBRF data (includes all NZ researchers) | | From 2018 survey of Australian Research Population | Gender balance by discipline measured in separate dataset [ref]; or from applicant pool if not available. | From applicant pool |
| **year** | 2006,  2012,  2018 | 2018 (using publications from 2013-18 inclusive) | 2010-2019 (inclusive) | 2019 | Pre-2013  Post-2013 |
| **Other demographic data available?** | Date of birth,  Rank (not used)  Ethnicity (sometimes, and incomplete) | same | No | No | Original study accounted for researcher age, but unavailable for our study |
| **Does each datapoint represent one individual?** | Yes | Yes | No. represents an applicant; one individual can apply for several grants. | No. Aggregated data reports # applicants, # successes in each country/discipline combination. | No. Aggregated data reports success rates By gender by time point in each discipline? |
| **Sample size** | 2006=4230  2012=6663  2018=7487 | 384 | Over 176,000 applicants aggregated into 440 datapoints (22 disciplines x 10 years x 2 genders) | Over 135,000 applicants aggregated into 333 data points ((8 disciplines x 28 countries x 2 genders) – missing data) | Over 23,000 applicants represented in 16 datapoints (4 disciplines x 2 time points x 2 genders) |
| **Independence** | High within year | same | Some individuals may have applied more than once | Some individuals may have applied more than once | Some individuals may have applied more than once |
| **Quotas/pre-allocation?** | No | No | No pre-allocation of funds to disciplines before applications scored and ranked. | Unknown | unknown |
| **Bibliometric data available?** | No | Per researcher:  # outputs,  # weighted outputs (divided by # of co-authors)  Mean field weighted citation index  Mean SNIP  Mean # citations/output | No | No | no |
